# Agreement of amyloid PET and CSF biomarkers for Alzheimer's disease on Lumipulse

**DOI:** 10.1002/acn3.50873

**Published:** 2019-08-28

**Authors:** Daniel Alcolea, Jordi Pegueroles, Laia Muñoz, Valle Camacho, Diego López‐Mora, Alejandro Fernández‐León, Nathalie Le Bastard, Els Huyck, Alicia Nadal, Verónica Olmedo, Frederic Sampedro, Victor Montal, Eduard Vilaplana, Jordi Clarimón, Rafael Blesa, Juan Fortea, Alberto Lleó

**Affiliations:** ^1^ Sant Pau Memory Unit, Department of Neurology Hospital de la Santa Creu i Sant Pau ‐ Biomedical Research Institute Sant Pau (IIB Sant Pau), Universitat Autònoma de Barcelona Barcelona Spain; ^2^ Centro de Investigación Biomédica en Red de Enfermedades Neurodegenerativas, CIBERNED Madrid Spain; ^3^ Nuclear Medicine Department Hospital de la Santa Creu i Sant Pau, Biomedical Research Institute Sant Pau, Universitat Autònoma de Barcelona Barcelona Spain; ^4^ Fujirebio Europe N.V. Gent Belgium; ^5^ Fujirebio Iberia, S.L. Barcelona Spain

## Abstract

**Objective:**

To determine the cutoffs that optimized the agreement between ^18^F‐Florbetapir positron emission tomography (PET) and A*β*1‐42, A*β*1‐40, tTau, pTau and their ratios measured in cerebrospinal fluid (CSF) on the LUMIPULSE G600II instrument, we quantified the levels of these four biomarkers in 94 CSF samples from participants of the Sant Pau Initiative on Neurodegeneration (SPIN cohort) using the Lumipulse G System with available ^18^F‐Florbetapir imaging.

**Methods:**

Participants had mild cognitive impairment (*n* = 35), AD dementia (*n* = 12), other dementias or neurodegenerative diseases (*n* = 41), or were cognitively normal controls (*n* *= *6). Levels of A*β*1‐42 were standardized to certified reference material. Amyloid scans were assessed visually and through automated quantification. We determined the cutoffs of CSF biomarkers that optimized their agreement with ^18^F‐Florbetapir PET and evaluated concordance between markers of the amyloid category.

**Results:**

A*β*1‐42, tTau and pTau (but not A*β*1‐40) and the ratios with A*β*1‐42 had good diagnostic agreement with ^18^F‐Florbetapir PET. As a marker of amyloid pathology, the A*β*1‐42/A*β*1‐40 ratio had higher agreement and better correlation with amyloid PET than A*β*1‐42 alone.

**Interpretation:**

CSF biomarkers measured with the Lumipulse G System show good agreement with amyloid imaging in a clinical setting with heterogeneous presentations of neurological disorders. Combination of A*β*1‐42 with A*β*1‐40 increases the agreement between markers of amyloid pathology.

## Introduction

Advances in the field of biomarkers have pushed forward a redefinition of Alzheimer's disease (AD) as a biological construct.^1^ Under this definition, the ATN classification system recognizes three general groups of biomarkers for AD: biomarkers of *β*‐amyloid plaques (A), biomarkers of fibrillar tau (T) and biomarkers of neurodegeneration or neuronal injury (N).^1^ These categories can be assessed by using different modalities, but those that are more widely implemented are cerebrospinal fluid (CSF) biomarkers and imaging techniques. Amyloid positron emission tomography (PET), CSF A*β*1‐42 and the ratio A*β*1‐42/A*β*1‐40 correspond to the “A” category, Tau PET and CSF pTau to the “T” category, whereas ^18^F‐Fluorodeoxyglucose‐PET, anatomical MRI and tTau are considered markers of the “N” category.^1^ Within the “A” category, the concordance between CSF A*β*1‐42 and amyloid PET imaging is high, but not perfect.^2^, ^3^, ^4^, ^5^, ^6^ In fact, the A*β*1‐42/A*β*1‐40 ratio has shown to have better agreement with amyloid PET imaging compared to levels of A*β*1‐42 alone.^4^, ^7^, ^8^, ^9^

In recent years, fully automated platforms have been developed for the analysis of CSF biomarkers. Recently, four CSF analytes (A*β*1‐42, A*β*1‐40, tTau and pTau) have been implemented on the fully automated Lumipulse G System, but there are no validated cutoffs for these four AD CSF biomarkers using this platform. Our aims were to determine for the first time the cutoffs that optimized the agreement between ^18^F‐Florbetapir PET and A*β*1‐42, A*β*1‐40, tTau, pTau and their ratios measured in CSF on the LUMIPULSE G600II instrument, and to evaluate the concordance between markers of the amyloid category.

## Methods

### Study participants

We included 94 participants from the Sant Pau Initiative on Neurodegeneration (SPIN cohort) recruited between November 2013 and September 2017 who had available CSF samples and ^18^F‐Florbetapir PET imaging (Table 1). The SPIN cohort is a multimodal research cohort for biomarker discovery and validation that includes participants with different neurodegenerative dementias, mild cognitive impairment and cognitively normal controls. All participants receive an extensive neurological and neuropsychological evaluation and undergo structural 3T brain MRI, blood extraction, and lumbar puncture for CSF biomarkers. A subset of participants also receives molecular imaging such as ^18^F‐Fluorodeoxyglucose‐PET, amyloid and/or Tau PET. More information on the SPIN cohort can be found at https://santpaumemoryunit.com/our-research/spin-cohort (Alcolea et al., submitted). All participants gave written consent, and the ethics committee of Hospital Sant Pau approved all procedures included in this study.

**Table 1:** Clinical and demographic characteristics of all participants and based on visual amyloid PET status.

	All participants	Amyloid positive	Amyloid negative	*P* value
*n* (%)	94 (100%)	59 (63%)	35 (37%)	–
Age, years	73.0 (7.6)	73.5 (7)	72.1 (8.5)	0.405^*^
Sex, female/male (% female)	50/44 (53%)	32/27 (54%)	18/17 (51%)	0.960^†^
APOEε4 +/− (% +)	56/37 (60%)	30/28 (52%)	7/28 (20%)	0.004^†^
MMSE score	24.7 (4.1)	24.1 (4.3)	25.7 (3.6)	0.052^*^
Time difference between amyloid PET and lumbar puncture, days	152 (86)	151 (78)	156 (100)	0.792^*^
Clinical diagnosis, *n* (%)				0.067^†^
Cognitively normal	6 (100%)	3 (50%)	3 (50%)	–
Mild cognitive impairment	35 (100%)	23 (66%)	12 (34%)	–
AD dementia	12 (100%)	11 (92%)	1 (8%)	–
Dementia with Lewy bodies	30 (100%)	18 (60%)	12 (40%)	–
Frontotemporal dementia	9 (100%)	3 (33%)	6 (67%)	–
Other diagnoses	2 (100%)	1 (50%)	1 (50%)	–
CSF biomarkers				
A*β*1‐42, pg/mL	745 (379)	608 (213)	985 (474)	<0.001^*^
A*β*1‐40, pg/mL	12252 (3944)	12782 (3954)	11360 (3816)	0.089^*^
tTau, pg/mL	566 (363)	667 (375)	397 (273)	<0.001^*^
pTau, pg/mL	93 (71)	114 (71)	58 (58)	0.001^*^
A*β*1‐42/A*β*1‐40	0.064 (0.028)	0.049 (0.015)	0.088 (0.027)	<0.001^*^
tTau/A*β*1‐42	0.95 (0.76)	1.17 (0.63)	0.57 (0.81)	<0.001^*^
pTau/A*β*1‐42	0.16 (0.15)	0.20 (0.12)	0.09 (0.16)	<0.001^*^

Unless otherwise specified, results are presented as mean (standard deviation).

MMSE, Mini‐mental state examination; AD, Alzheimer's disease; CSF, cerebrospinal fluid; PET, positron emission tomography.

*P*‐values were calculated by comparing amyloid–positive and amyloid–negative participants using Welch two‐sample *t*‐test (*) or Fisher's exact test (^†^).

### CSF samples acquisition and analysis

CSF samples were collected in 10 mL polypropylene tubes (Sarstedt, Ref#62.610.018) and transferred to the Sant Pau Memory Unit's laboratory where they were processed within the first 2 h after acquisition. After centrifugation (2000*g* × 10 min, 4°C), volumes of 0.5 mL of CSF were aliquoted into polypropylene tubes (Sarstedt, Ref#72.694.007) and stored at −80°C until analysis.

On the day of the analysis, samples were thawed at room temperature and the tubes were vortexed for 5–10 sec. To avoid the effect of multiple freeze‐thaw cycles, aliquots used in this study had not been thawed before. A*β*1‐42, A*β*1‐40, tTau and pTau were quantified directly from the storage tubes containing 0.5 mL of CSF using the Lumipulse G *β*‐Amyloid 1‐42, *β*‐Amyloid 1‐40, Total Tau and pTau 181 assays on LUMIPULSE G600II automated platform (Fujirebio) and following the manufacturer's instructions. We used an adapter to fit the tubes in the equipment. We used the same batch of reagents for each biomarker throughout the study, and for each sample, we measured all four analytes from the same aliquot and in the same run. The platform was configured to start the analysis with A*β*1‐42, followed by A*β*1‐40, tTau and pTau. Buffer‐based quality control testing was performed at the beginning of each test day to ensure that all measured values of each control level (low, medium and high) were within the target ranges.

The results of the Lumipulse G *β*‐Amyloid 1‐42 presented in this study have been standardized according to certified reference material developed by the International Federation of Clinical Chemistry and Laboratory Medicine as recommended by their working group for CSF proteins.^10^ Briefly, values of the calibration standards of the LUMIPULSE G600II were adapted to the certified reference material resulting in an adjustment of concentrations that was linearly proportional throughout the range. The aim of standardization to certified reference material is to harmonize immunoassays of A*β*1‐42 to make results comparable across different platforms.

A*β*1‐42, A*β*1‐40, tTau and pTau levels in CSF from participants of this study had been measured previously using other immunoassays (INNOTEST *β*‐AMYLOID_(1‐42)_, INNOTEST hTAU Ag, and INNOTEST PHOSPHO‐TAU_(181P)_, Fujirebio Europe; and High Sensitivity Human Amyloid *β*40, Merck‐Millipore), and these results were available in our database for their comparison with the LUMIPULSE analyses.^11^, ^12^, ^13^, ^14^, ^15^

The personnel involved in the CSF analyses for this study were blinded to the clinical diagnosis and to previous biomarker determinations.

### Amyloid‐PET imaging acquisition, visual assessment and quantitative analysis

All participants underwent amyloid PET imaging with ^18^F‐Florbetapir as described elsewhere.^13^ PET data were acquired using a Philips Gemini TF scan 50 min after injection of 370mBq of ^18^F‐Florbetapir. After obtaining the transmission data, brain PET dynamic acquisition was performed (2 × 5 min frames). The reconstruction method was iterative (LOR RAMBLA, three iterations and 33 subsets) with a 128 × 128 image size, 2 mm pixel size and slice thickness.

Three expert readers (V.C., D.L‐M. and A.F‐L.) blind to clinical diagnosis and to CSF biomarkers visually rated all PET scans. Following manufacturer's protocol, scans were classified as “positive” when one or more areas showed increased cortical gray matter signal resulting in reduced or absent contrast between gray matter and white matter. Scans were classified as “negative” when the contrast between gray matter and white matter was clear. Final classification as “positive” or “negative” was decided upon agreement of at least two of three readers. Mean inter‐reader overall agreement was 88.4% (Min = 87.0%, Max = 90.2%).

We also quantified amyloid deposition. Each participant's PET scan was spatially normalized to a MNI152 ^18^F‐Florbetapir template using a linear and nonlinear transformation.^16^ Mean ^18^F‐Florbetapir uptake was measured across frontal, lateral parietal, lateral temporal and anterior/posterior cingulate. Then, the ^18^F‐Florbetapir standardized uptake value ratio (SUVR) map was extracted using the whole cerebellum as reference.^17^ The PET scans of five participants were not suitable for ^18^F‐Florbetapir quantification and excluded of quantitative analyses.

### Statistical analysis

We performed receiver operating characteristic (ROC) analysis for A*β*1‐42, A*β*1‐40, tTau, pTau and the ratios A*β*1‐42/A*β*1‐40, tTau/A*β*1‐42 and pTau/A*β*1‐42 to calculate areas under the curve (AUC) with 95% confidence intervals (DeLong). We compared ROC curves by two‐sided bootstrapping with 2000 replications. For biomarkers and ratios that showed AUC higher than 0.70, we determined positive percent agreement (PPA or sensitivity) and negative percent agreement (NPA or specificity) and calculated optimal cutoffs maximizing their Youden J index (PPA + NPA − 1). We calculated overall percent agreement (OPA) between CSF biomarker cutoffs and amyloid PET visual interpretation as the sum of participants classified as “positive” or as “negative” by both modalities over the total number of participants. We also analyzed the correlation of CSF biomarkers with global amyloid accumulation by fitting quadratic models and calculated the agreement of CSF biomarkers cutoffs with the PET scans quantification status applying a previously described SUVR cutoff of 1.11.^17^ Level of significance was set at *α* = 0.05. We used Analyse‐it^®^ statistical software for the selection of optimal cutoffs and packages “car” (v.3.0‐3, Fox&Weisberg 2019), “pROC” (v.1.15.0, Robin et al 2011), “grid” (R core team 2019) and “ggplot2” (v.3.1.1, Wickham 2016) as implemented in R statistical software (v 3.6.0) for plots and statistical analyses.

## Results

### Study participants

We included 94 participants in the study. Table 1 summarizes demographic characteristics and biomarker results. There were no differences in age or sex between both groups. As expected, the amyloid–positive group had a higher proportion of *APOEε4* carriers compared to the amyloid–negative group (52% and 20%, respectively; *P* = 0.004).

### Quantification of A*β*1‐42, A*β*1‐40, tTau and pTau concentrations on the LUMIPULSE G600II

We measured A*β*1‐42, A*β*1‐40, tTau and pTau levels simultaneously on the Lumipulse G System. Their levels in the overall study population ranged from 315 to 2280 pg/mL for A*β*1‐42, 4585 to 25925 pg/mL for A*β*1‐40, 141 to 1902 pg/mL for tTau, and 18 to 340 pg/mL for pTau.

The analyses were divided over three calibration runs on the LUMIPULSE G600II, and the calibration status was valid for all samples. Mean interassay coefficients of variation are displayed in Figure S1.

Most CSF samples included in this study had previously been analyzed using other immunoassays, and their results were available in our database. Although these historic results were obtained in the context of routine clinical assessment by using different batches, and therefore a side‐to‐side precision analysis could not be performed, we explored their correlation with the Lumipulse G quantifications. The Lumipulse G assays for A*β*1‐42, tTau and pTau showed very high correlation with values previously measured with Fujirebio's INNOTEST ELISA (Pearson's r of 0.94, 0.95 and 0.95, respectively, all *P* < 0.001). The Lumipulse G assay for A*β*1‐40 showed moderate correlation with values measured with Merck‐Millipore's ELISA (Pearson's *r* of 0.76, *P* < 0.001). On the Lumipulse G system, tTau and pTau were highly correlated (Pearson's *r* of 0.98, *P* < 0.001).

### Agreement between ^18^F‐Florbetapir visual status and CSF biomarkers

**Figure 1:**
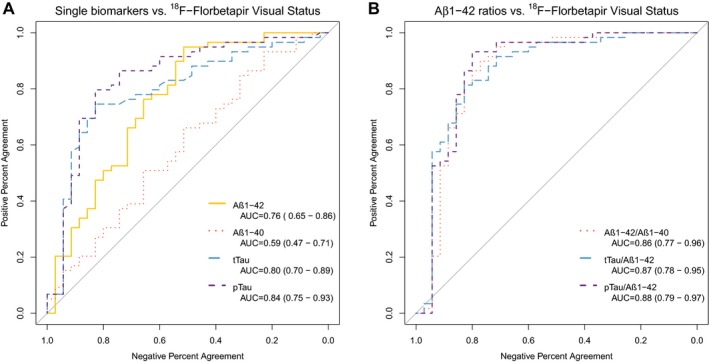
Receiver operating characteristic analysis of individual (A) and combined (B) CSF biomarkers' diagnostic utility to detect amyloid visual status. AUC, Area under the curve.

As displayed in Figure 1A, of individual biomarkers, tTau and pTau had the highest accuracy and showed AUC of 0.80 (95%CI 0.70–0.89, *P* < 0.001) and 0.84 (95%CI 0.75–0.93, *P* < 0.001), respectively. A*β*1‐42 had fair accuracy with an AUC of 0.76 (95%CI 0.65–0.86, *P* < 0.001) and A*β*1‐40 alone was not useful for the detection of the visual status of amyloid scans (AUC 0.59; 95%CI 0.47–0.71, *P* = 0.134).

Figure 1B shows that the combination of A*β*1‐42 with a second analyte resulted in significant increases of accuracy. The AUC of A*β*1‐42/A*β*1‐40 was 0.86 (95%CI 0.77–0.96, *P* < 0.001), higher than that of A*β*1‐42 alone (*D* = −2.5; *P* = 0.01) or A*β*1‐40 alone (*D* = −4.0; *P* < 0.001). tTau/A*β*1‐42 had an AUC of 0.87 (95%CI 0.78–0.95, *P* < 0.001), higher than that of tTau (*D* = −2.2; *P* = 0.03), and pTau/A*β*1‐42 had an AUC of 0.88 (95%CI 0.79–0.97, *P* < 0.001), higher than that of pTau alone (*D* = −1.9; *P* = 0.05). There were no significant differences in the AUC of the three ratios, and combining a third biomarker in the ratios did not improve their accuracy (data not shown).

### CSF biomarker cutoffs based on visual interpretation of amyloid status

**Figure 2:**
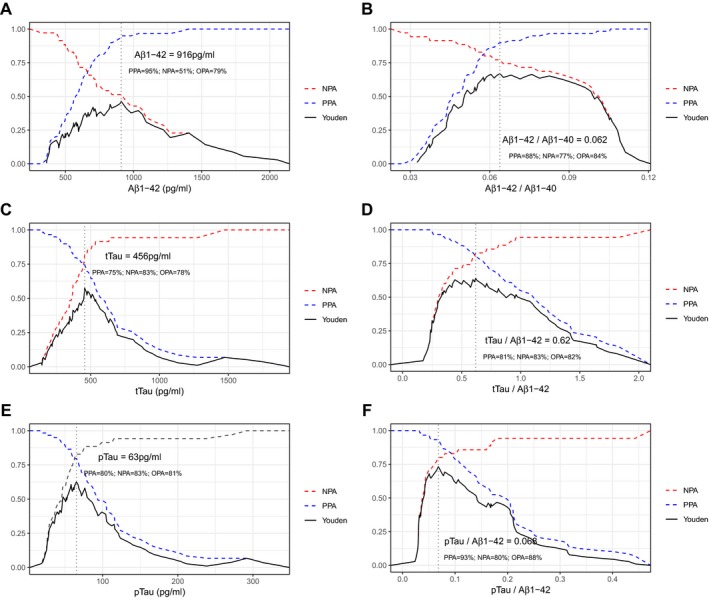
Accuracy of all possible cutoff levels of individual (A, C, E) and combined (B, D, F) CSF biomarkers. Only those biomarkers that yielded areas under the curve above 0.70 and their ratios with A*β*1‐42 are shown. Vertical dotted lines indicate cutoffs with maximum Youden J index. PPA, Positive Percent Agreement; NPA, Negative Percent Agreement; OPA, Overall Percent Agreement.

For those biomarkers and ratios that showed AUC higher than 0.70, we used ROC analysis to obtain PPA, NPA and OPA for all possible cutoffs. As displayed in Figure 2, in the case of single biomarkers, A*β*1‐42, tTau and pTau, the selection was based on clear Youden peaks at 916, 456 and 63 pg/mL, respectively. For the ratios A*β*1‐42/A*β*1‐40, tTau/A*β*1‐42 and pTau/A*β*1‐42, plots showed *plateau* stages indicating that a wide range of cutoffs yielded similar Youden indices. Best cutoffs for ratios were 0.062 for A*β*1‐42/A*β*1‐40, 0.62 for tTau/A*β*1‐42, and 0.068 for pTau/A*β*1‐42.

**Figure 3:**
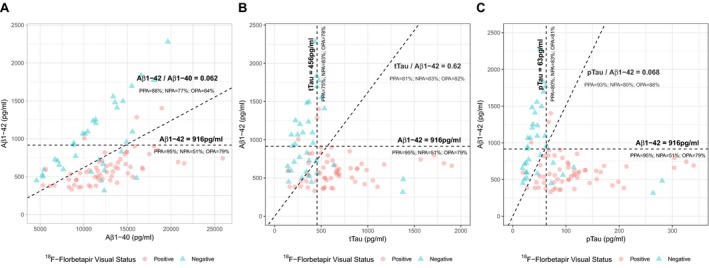
Agreement of visual amyloid status with single and combined CSF biomarkers. Panels A, B and C display scatterplots of CSF biomarker levels. Dashed lines indicate cutoffs that yielded maximum Youden J Index in the receiver operating characteristic analysis for each biomarker or ratio. PPA, Positive Percent Agreement; NPA, Negative Percent Agreement; OPA, Overall Percent Agreement.

Figure 3 displays the agreement between visual status of ^18^F‐Florbetapir and CSF biomarker cutoffs. For A*β*1‐42, tTau and pTau, the OPA values between visual status and CSF biomarkers status were 79%, 78% and 81%, respectively. The ratio of A*β*1‐42 with A*β*1‐40, tTau and pTau increased the OPA to 84%, 82% and 88%, respectively.

### Markers of amyloid and importance of assessing a second biomarker

Within each CSF biomarker status, the proportion of positive amyloid scans varied when a second biomarker or ratio was taken into account. Figure S2 shows the proportion of positive amyloid scans within each combination of two CSF biomarkers or ratios, and illustrates the importance of considering a second biomarker. Of all participants with low CSF levels of A*β*1‐42, regardless of the A*β*1‐42/A*β*1‐40 ratio status, 77% (56 out of 73) had a positive amyloid scan. This proportion increased to 87% (52 out of 60) within this group when the A*β*1‐42/A*β*1‐40 ratio was also low but decreased to 31% (four out of 13) when the A*β*1‐42/A*β*1‐40 ratio was high. In the group of participants with high CSF levels of A*β*1‐42, the impact of considering the A*β*1‐42/A*β*1‐40 status had no effect, as in all participants within this group the A*β*1‐42/A*β*1‐40 ratio was also high. These results highlight the importance of using the A*β*1‐42/A*β*1‐40 ratio over A*β*1‐42 alone in the assessment of brain amyloidosis, especially in patients with low CSF levels of A*β*1‐42.

### Agreement of CSF cutoffs with amyloid quantification

**Figure 4:**
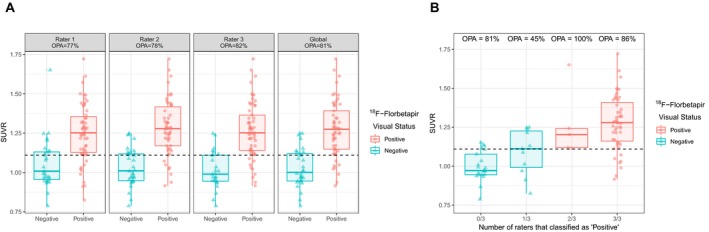
Agreement between raters' visual classification and amyloid quantification. OPA, Overall Percent Agreement; SUVR, Standardized Uptake Value Ratio. Panel A shows the agreement between amyloid quantification and rater's individual and global visual assessments. Panel B shows the agreement between amyloid quantification and visual assessment stratified by the number of raters that assessed scans as positive.

We next processed amyloid PET scans to obtain quantification values of amyloid deposition. In our study, the previously validated SUVR value of 1.11,^17^ showed 83% PPA, 76% NPA, and 81% OPA with visual classification. As displayed in Figure 4, scans that were divergently classified as “negative” or “positive” by one of the three raters showed intermediate SUVR values compared to scans that were unanimously classified.

**Figure 5:**
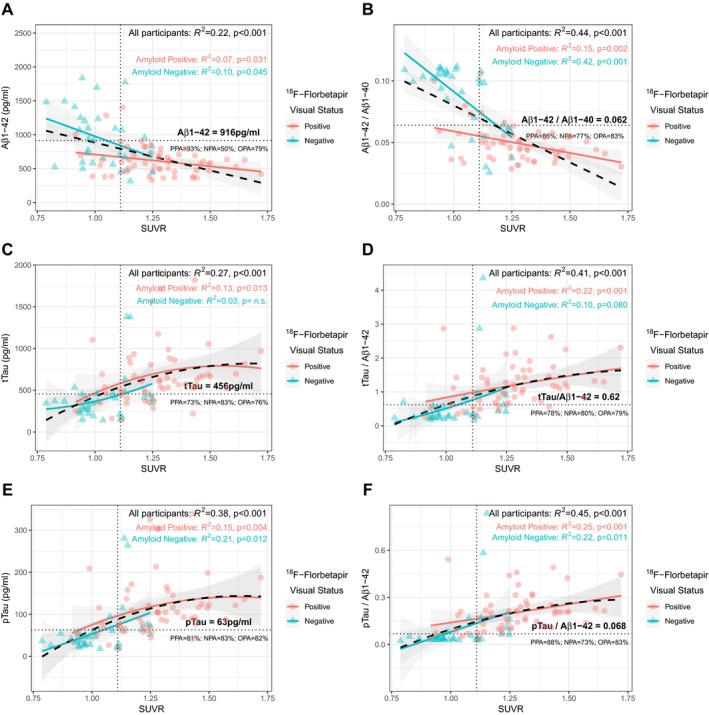
Scatterplots and correlations of amyloid quantification values with individual biomarkers (A, C, E) and ratios (B, D, F). Correlation between SUVR values and CSF biomarkers was assessed by fitting quadratic models for all participants (black) and after stratifying by visual amyloid status (red and green). Shaded areas indicate 95% confidence intervals. Dashed vertical lines indicate the SUVR cutoff of 1.11 as in Landau et al. Horizontal lines correspond to cutoffs for each CSF biomarker and ratio. PPA, NPA and OPA values correspond to the agreement between amyloid quantification and CSF biomarkers. PPA, Positive Percent Agreement; NPA, Negative Percent Agreement; OPA, Overall Percent Agreement; SUVR, Standardized Uptake Value Ratio.

As seen in Figure 5, the agreement of CSF cutoffs with amyloid PET quantification was similar to that with visual classification for all biomarkers. We studied the correlation between each CSF biomarker and global amyloid accumulation by fitting quadratic models. In these models, the adjusted coefficients of determination (*R^2^*) were higher for all ratios compared to individual biomarkers. The combination of A*β*1‐42 with A*β*1‐40 increased the *R^2^* value to 0.44 (*P* < 0.001), indicating that this ratio reflects the amyloid deposition better than A*β*1‐42 alone. Stratified analysis by ^18^F‐Florbetapir visual status showed lower *R^2^* values for all biomarkers, which suggests that the correlation observed between CSF biomarkers and amyloid PET quantification is partially mediated by an amyloid‐status effect. In this stratified analysis, the highest correlation of SUVR values was found with the A*β*1‐42/A*β*1‐40 ratio in the amyloid–negative group (*R^2^* = 0.42; *P* < 0.001).

## Discussion

In our study, we determined cutoffs for four CSF biomarkers for AD (A*β*1‐42, A*β*1‐40, tTau and pTau) and their ratios measured on the fully automated LUMIPULSE G600II platform to optimize their concordance with ^18^F‐Florbetapir PET. We calibrated A*β*1‐42 levels to certified reference material, recently developed to harmonize immunoassays across different platforms, and found that the ratios A*β*1‐42/A*β*1‐40, tTau/A*β*1‐42 and pTau/A*β*1‐42 had better diagnostic agreement with visual assessment of amyloid scans than single biomarkers. As a marker of amyloid pathology, the A*β*1‐42/A*β*1‐40 ratio had higher agreement with amyloid PET visual status and showed better correlation with amyloid load quantification compared to A*β*1‐42 alone.

The agreement between amyloid imaging and AD CSF biomarkers has previously been studied by using other automated immunoassays.^3^, ^4^, ^8^ Our results are in line with previous studies showing a good overall agreement between amyloid imaging and AD CSF biomarkers, higher for ratios than for single analytes.^3^, ^4^ However, specific cutoff points for CSF biomarkers differ between these studies, and several methodological differences can explain these discrepancies. First, preanalytical conditions, such as the type of collection and storage tubes, are different between studies, and these factors are known to have a great impact on the absolute values of CSF biomarkers, especially for A*β*1‐42.^18^, ^19^ Second, some analytical particularities for each immunoassay and platform used in these studies (specificity of the antibodies, time of incubation) result in diverse CSF biomarker measures. Calibration of all automated platforms to certified reference material, currently underway, will minimize this issue in the future. Likewise, differences in the affinity of PET radiotracers (^11^C‐Pittsburg compound B, ^18^F‐Flutemetamol or ^18^F‐Florbetapir) can lead to disparities in the selection of cutoffs. Third, the composition of the populations was not the same across studies. Schindler et al. analyzed data from community–dwelling volunteers,^4^ whereas Janelidze et al. obtained their results from patients with mild cognitive impairment and subjective cognitive decline from the BioFINDER cohort.^8^ Hansson et al. studied CSF of participants from ADNI and BioFINDER cohorts, that included cognitively normal volunteers, patients with mild cognitive impairment and patients with AD dementia.^3^ In our study, we additionally included patients with other dementias or neurodegenerative diseases, which might reflect more realistically the application of biomarkers in daily clinical practice. As in a number of other studies, the cutoffs in our study were selected by maximization of Youden J index. This approach balances sensitivity and specificity and is equivalent to maximize accuracy for a pre‐test disease prevalence of 50%.^20^ However, other strategies might be useful in certain clinical scenarios.^21^ For instance, for screening purposes, it might be helpful to apply cutoffs with high sensitivity, even when their specificity is lower. For patients with clinically challenging diagnoses or in clinical trials, however, high specificity might be preferable. Other possible approaches include the sequential application of biomarker cutoffs.

The LUMIPULSE G600II has incorporated the possibility of measuring CSF levels of A*β*1‐40. In previous studies, and ours, this biomarker alone was not useful for the detection of brain amyloid.^4^, ^7^, ^9^ Both A*β*1‐42 and the A*β*1‐42/A*β*1‐40 ratio are included in the “A” category of the ATN classification system together with amyloid PET, but in line with other studies,^7^, ^9^, ^22^, ^23^ we found that the A*β*1‐42/A*β*1‐40 ratio had better agreement with visual amyloid status and higher correlation with brain amyloid quantification. Our results are in line with previous literature that suggests that the use of the A*β*1‐42/A*β*1‐40 ratio could compensate individual differences in amyloid precursor protein processing that otherwise might lead to false positive or false negative A*β*1‐42 CSF levels.^24^, ^25^ This information adds to the fact that using the A*β*1‐42/A*β*1‐40 ratio has proven to partially mitigate the effect of some preanalytical confounders that have been described to alter the results of amyloid levels.^26^, ^27^, ^28^ Altogether, our data suggest that the use of the A*β*1‐42/A*β*1‐40 ratio would be more reliable in clinical practice than A*β*1‐42 alone as a marker of amyloidosis and that this combination should be used in routine.

The main strength of our study is that four AD CSF biomarkers (A*β*1‐42, A*β*1‐40, tTau and pTau) measured simultaneously with the automated Lumipulse G System were compared for the first time to ^18^F‐Florbetapir PET to calculate amyloid–based cutoffs. In addition, this is, to our knowledge, the first study to present A*β*1‐42 levels that have been standardized to certified reference material, recently developed to harmonize immunoassays across different platforms. Standardized values will make our study more easily comparable to future studies. Moreover, to avoid possible sources of variability, we followed homogeneous CSF preanalytical and analytical procedures and used the same batch of reagents for all measurements. Also, the inclusion of participants with neurodegenerative diseases outside the AD spectrum provides a more realistic application of biomarkers in daily clinical practice.

However, we are aware of some limitations. We did not test the effect that deviations from our preanalytical protocol would have on the final cutoffs, and therefore, the cutoffs that we report should be taken cautiously under other operating procedures. Some of the clinical categories in our study included a small number of participants. Although we used amyloid positivity/negativity as the gold standard in our study, the composition of the sample in terms of clinical diagnosis might be relevant for the interpretation and applicability of our results. Additionally, only very few participants had additional Tau imaging and/or ^18^F‐Fluorodeoxyglucose‐PET, and we could not compare the agreement of CSF pTau and tTau with molecular imaging markers of the “T” and the “N” categories of the ATN classification. Likewise, as participants in this study are part of a living cohort, neuropathological confirmation is not available at this moment.

In this study, we found that the A*β*1‐42 ratios to A*β*1‐40, tTau and pTau in CSF show a good agreement with amyloid visual status and that the A*β*1‐42/A*β*1‐40 ratio had better correlation with the amount of amyloid burden compared to A*β*1‐42 alone. The understanding of the agreement between CSF biomarkers and amyloid imaging is crucial to identify situations in which these two modalities might not be interchangeable. This information has to be taken into consideration both in the diagnostic assessment in clinical practice and in the selection of participants in clinical trials.

## Conflict of Interest

D.A. participated in advisory boards from Fujirebio‐Europe and received speaker honoraria from Fujirebio‐Europe, Nutricia and from Krka Farmacéutica S.L. R.B. participated in advisory boards from Lilly and Nutricia, and he received speaker honoraria and travel funding from Novartis and Nutricia. A.L. participated in advisory boards from Fujirebio‐Europe, Nutricia, Biogen, and received speaker honoraria from Lilly. N.LB. and E.H. are employed by Fujirebio Europe. N.V. A.N. and V.O. are employed by Fujirebio Iberia, S.L.

## Supporting information


**Figure S1.** Interassay coefficients of A*β*1‐42 (A), A*β*1‐40 (B), tTau (C) and pTau (D).
**Figure S2.** Agreement of visual amyloid PET with the combination of two CSF biomarkers or ratios.
