# Supplementary figures and images for: Agreement of amyloid PET and CSF biomarkers for Alzheimer's disease on Lumipulse
Source: Ann Clin Transl Neurol. 2019 Aug 28;6(9):1815–24. doi: 10.1002/acn3.50873 (PMC6764494; doi:10.1002/acn3.50873)

**A**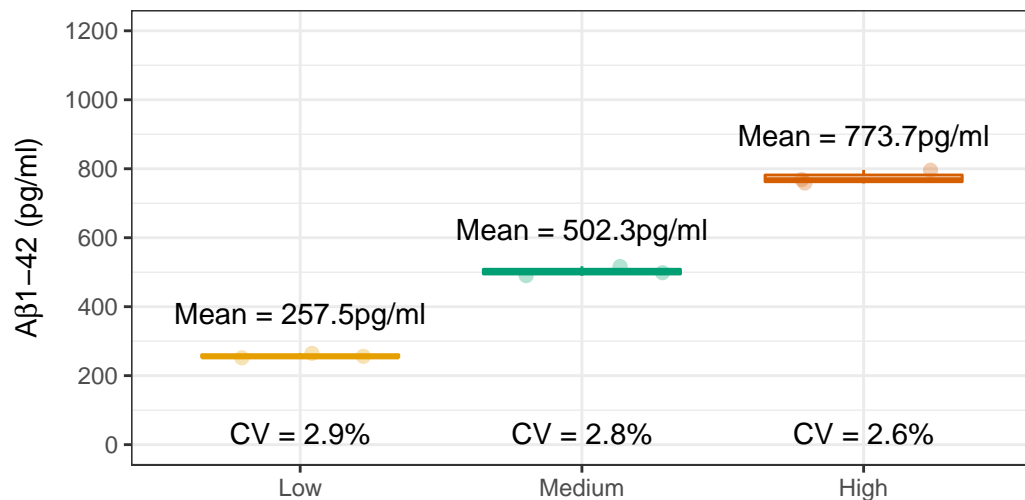**B**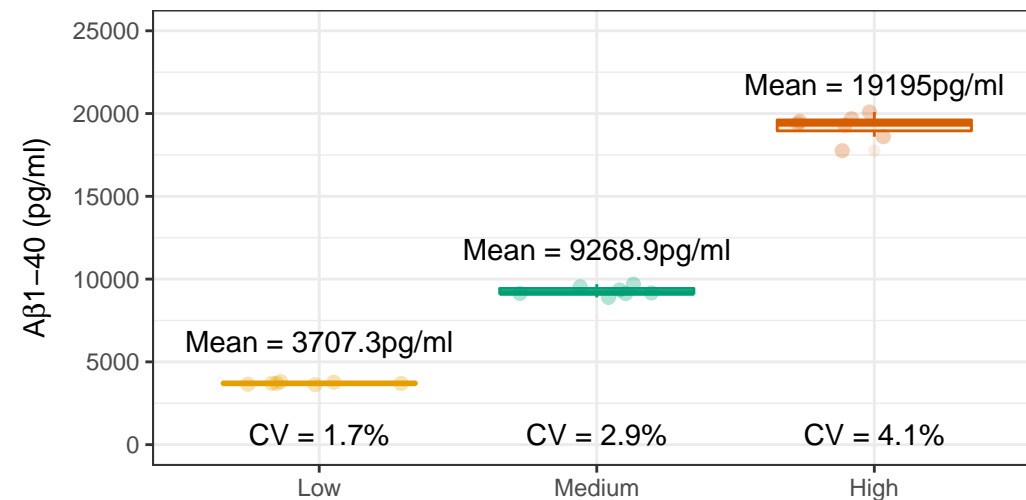**C**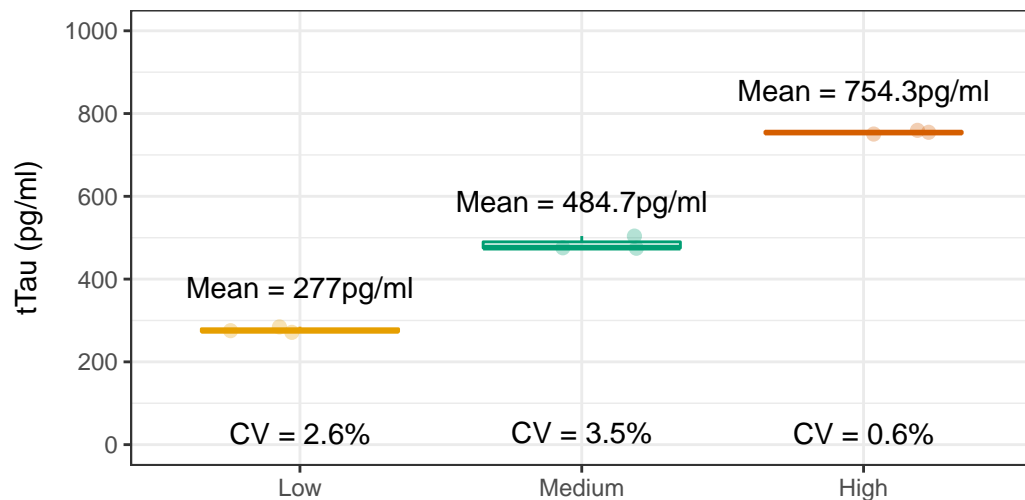**D**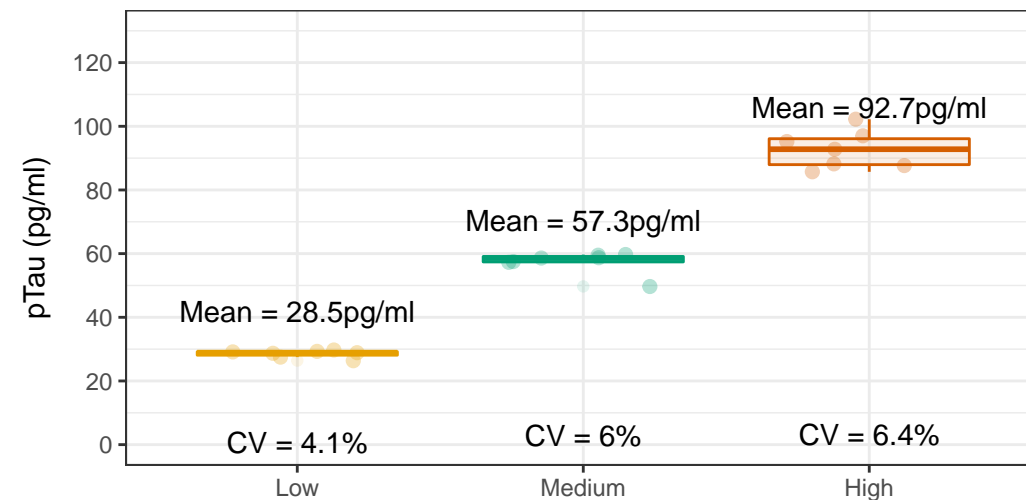

A – Amyloid PET positivity

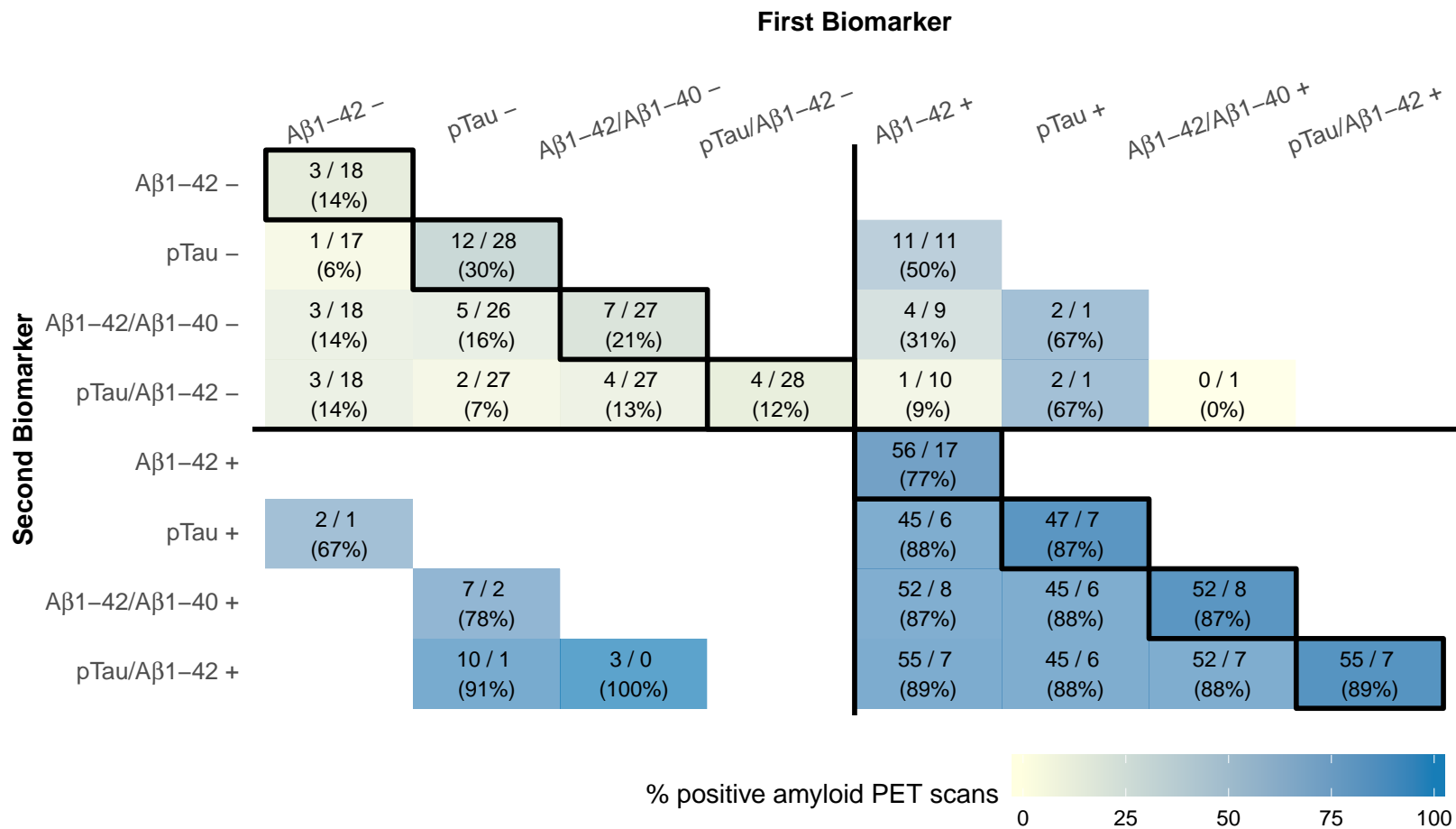

B – Amyloid PET quantification

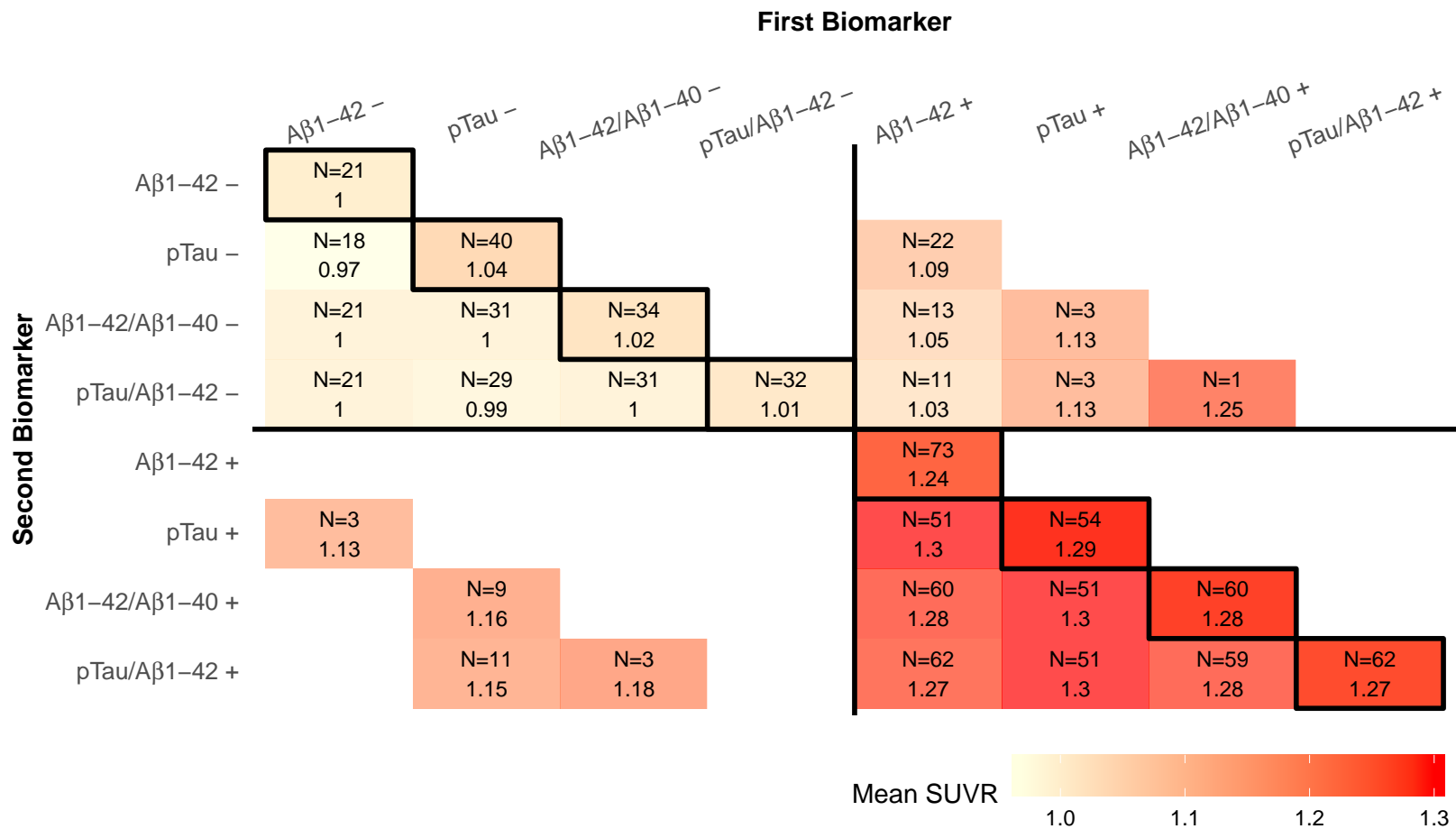

Supplement: Supplementary file 1 — Figure S1. Interassay coefficients of Aβ1‐42 (A), Aβ1‐40 (B), tTau (C) and pTau (D). Figure S2. Agreement of visual amyloid PET with the combination of two CSF biomarkers or ratios. [file ACN3-6-1815-s001.pdf]
